# Association between monocyte-to-HDL ratio and Fazekas-scaled white matter hyperintensities in migraine patients: a cross-sectional MRI-based study

**DOI:** 10.3389/fneur.2025.1664839

**Published:** 2025-12-15

**Authors:** Aydın Talip Yıldoğan, Ramazan Şencan, Gizem Türker Yıldoğan, Gözde Öngün, Bekir Sıtkı Said Ulusoy, Fettah Eren, Abdurrahman Neyal

**Affiliations:** 1 Department of Neurology, Faculty of Medicine, Nigde Omer Halis Demir University, Nigde, Türkiye; 2 Neurology Clinic, Gaziantep 25 Aralık State Hospital, Gaziantep, Türkiye; 3 Department of Biochemistry, Faculty of Medicine, Gaziantep University, Gaziantep, Türkiye; 4 Neurology Clinic, İstinye University Hospital, Istanbul, Türkiye; 5 Radiology Clinic, Gaziantep City Hospital, Gaziantep, Türkiye; 6 Department of Neurology, Faculty of Medicine, Selcuk University, Konya, Türkiye; 7 Department of Neurology, Faculty of Medicine, GIBTU University, Gaziantep, Türkiye

**Keywords:** migraine, white matter hyperintensities, Fazekas scale, monocyte-to-HDL ratio (MHR), inflammation

## Abstract

**Background:**

This study aimed to investigate the association between the monocyte-to-high density lipoprotein (HDL) cholesterol ratio (MHR) and the presence and severity of white matter hyperintensities (WMHs) in patients with migraine.

**Methods:**

A total of 153 patients diagnosed with migraine and 90 age- and sex-matched healthy controls were included. Serum monocyte and HDL levels, and MRI findings were evaluated. WMHs severity was graded using the Fazekas scale. Serum parameters were compared and evaluated between the patient and control groups. In addition, the relationship between migraine clinical characteristics and MHR levels was examined.

**Results:**

MHR values were significantly higher in migraine patients than in controls (*p* < 0.001). Higher Fazekas scores were associated with increased MHR and monocyte levels, and decreased HDL levels. Receiver operating characteristic (ROC) analysis indicated that an MHR > 13.37 predicted the presence of WMHs with 88.50% sensitivity and 98% specificity (AUC = 0.918, *p* < 0.001).

**Conclusion:**

MHR appears to be a promising inflammatory biomarker reflecting cerebral microvascular changes in migraine patients, even among young adults. These results support the contribution of low-grade systemic inflammation to migraine-associated white matter lesions.

## Introduction

Migraine is a prevalent primary headache disorder influenced by both genetic and environmental factors. It is now widely accepted as a neurovascular condition, with activation of the trigeminovascular pain pathway that has a central role in its pathophysiology (1, 2).

Although brain imaging is typically normal in most migraine patients, white matter hyperintensities (WMHs) are frequently observed, especially on magnetic resonance imaging (MRI) (3, 4). WMHs are among the most common structural abnormalities in the white matter, usually detecting as small, punctate, non-mass lesions on T2-weighted and fluid-attenuated inversion recovery (FLAIR) sequences (5).

The exact pathogenesis of WMHs remains unclear, but they are believed to result from chronic ischemic processes linked to small vessel disease. In addition, WMHs are known to increase with aging (6, 7). Several recent studies have detected that WMHs are significantly more prevalent in migraine patients compared to healthy individuals (3, 4, 8). Although migraine subtype, disease duration, and attack frequency have been suggested as factors that may contribute to the occurrence and burden of WMH in migraine patients, the findings are not conclusive (3, 9).

Proposed mechanisms underlying WMH formation in migraine include regional hypoperfusion, ischemic microvascular injury, hypercoagulability, and endothelial dysfunction (10, 11). Oxidative stress, present during both ictal and interictal periods, may also contribute to WMH development (3, 12).

The monocyte-to-HDL cholesterol ratio (MHR) has recently been reported as a novel biomarker of inflammation and endothelial dysfunction. Higher MHR indicates a pro-inflammatory and pro-atherogenic state and it is associated with various conditions such as coronary artery disease, ischemic stroke, and schizophrenia (13–15). Based on the relationship between MHR and vascular dysfunction, it has been suggested that MHR may be a potential biomarker in migraine. However, MHR is not only associated with migraine; it is also linked to systemic inflammatory and cardiometabolic states, including insulin resistance and increased cardiovascular risk in rheumatoid arthritis and the inflammatory processes observed in gout (16, 17). It was reported that there was an association between MHR and WMHs in migraine patients, and that a positive correlation was also detected with Visual Analog Scale (VAS) and Migraine Disability Assessment Scale (MIDAS) scores (18). Similarly, Increased MHR was significantly associated with cerebral small vessel disease markers, including WMHs, lacunes, and microbleeds (19).

Although the Fazekas scale is widely used for semiquantitative WMH assessment few studies have explored the relationship between MHR and WMH in migraine populations (5). WMHs severity using the Fazekas classification was evaluated in our study. In addition, the relationship between this score and serum parameters were evaluated in migraine.

This study aimed to evaluate the association between MHR and WMH severity using Fazekas scale in patients with migraine. We sought to elucidate the inflammatory and vascular mechanisms contributing to migraine pathophysiology.

## Materials and methods

### Study design and population

This cross-sectional observational study was conducted from May 1, 2024, to November 1, 2024, at the Neurology Department of Dr. Ersin Arslan Training and Research Hospital. A total of 153 patients diagnosed with migraine according to the International Headache Society (IHS) criteria and 90 healthy controls were included (20). Inclusion criteria for this study were age between 18 and 65 years, anddiagnosis of migraine based on IHS criteria. Exclusion criteria were determined as history of hypertension, diabetes, stroke, or cardiovascular disease; renal, endocrine, or metabolic disorders; central nervous system (CNS) disease or malignancy; smoking; pregnancy, alcohol or substance use; and the last inability to complete assessments due to sociocultural or cognitive limitations. The control group was recruited from hospital volunteers during the same study period. Participants with a confirmed migraine diagnosis were included in the migraine group, and those with a migraine diagnosis were excluded from the control group. Beyond this distinction, the same inclusion and exclusion criteria were applied to both groups. Controls were matched with migraine patients by gender, age and body mass index (BMI). Most of the controls presented with nonspecific, mild complaints such as tension-type headache or transient dizziness that did not meet migraine criteria, and subsequent evaluations revealed no underlying organic pathology.

### Clinical and demographic assessment

All migraine patients completed a structured headache questionnaire and the MIDAS scale (21). Clinical data included headache type, duration, frequency, and aura status. Control subjects were matched by age and gender.

### Neuroimaging protocol

All participants underwent brain MRI using a 1.5 Tesla scanner (Siemens, Germany). WMHs were evaluated on axial FLAIR sequences. Imaging was interpreted by a neuroradiologist blinded to the clinical and laboratory data. WMH severity was graded using the Fazekas scale. Fazekas scalewere grouped as Fazekas 0: No lesions; Fazekas 1: Mild lesions; Fazekas 2: Moderate lesions; Fazekas 3: Large confluent areas (5).

### Serum assessment

Venous blood samples were collected to determine: All these parameters were detected monocyte count (cells/μL), HDL cholesterol (mg/dL) and MHR was calculated with formulation as (monocyte count/HDL). Additional parameters included complete blood count, C reactive protein (CRP), glucose, triglycerides, and low-density lipoprotein (LDL) cholesterol.

### Statistical analysis

Data were analyzed using SPSS version 23.0 (IBM Corp., Armonk, NY, USA) and MedCalc software. Continuous variables were expressed as mean ± standard deviation or median (interquartile range), and categorical variables as counts and percentages. Normality was assessed using the Shapiro–Wilk test. The Mann–Whitney U and Kruskal-Wallis tests were used for between-group comparisons. Chi-square tests were used for categorical data. Receiver operating characteristic (ROC) curves analyses were constructed for MHR, and optimal cut-off values were calculated. Binary and multinomial logistic regression analyses were performed to evaluate the impact of variables on WMH presence and severity. A *p*-value <0.05 was considered statistically significant.

## Results

### Clinical and laboratory characteristics

There were no significant differences between migraine patients (n = 153) and healthy controls (n = 90) in terms of age, sex, BMI, lymphocyte and neutrophil counts, glucose, LDL, hemoglobin, platelet count, or CRP levels (*p* > 0.05). However, monocyte count, HDL, MHR, triglycerides, and WBC levels showed significant differences (*p* < 0.05). Migraine patients had higher monocyte counts, MHR, triglyceride levels, and WBCs, while HDL levels were higher in the control group (Table 1).

**Table 1:** Clinical and laboratory characteristics of patients and controls.

Parameter	Migraine group (*n* = 153)	Control group (*n* = 90)	*p*-value
Age (years)	29.75 ± 5.30	29.02 ± 5.27	0.328
Females (n:186)	29.99 ± 5.49 **(n:117)**	28.64 ± 5.33 **(n:69)**	0.086
Males (n:57)	28.97 ± 4.61 **(n:36)**	30.29 ± 4.98 **(n:21)**	0.245
BMI (kg/m^2^)	24.38 ± 2.62	24.41 ± 2.55	0.883
Females	24.32 ± 2.6	24.4 ± 2.63	0.826
Males	24.57 ± 2.72	24.45 ± 2.34	0.888
Lymphocyte (10^3^/μL)	2.31 ± 0.96	2.38 ± 0.98	0.554
Females	2.3 ± 0.94	2.35 ± 0.98	0.717
Males	2.37 ± 1.03	2.5 ± 0.97	0.741
Neutrophil (10^3^/μL)	4.52 ± 0.85	4.43 ± 0.86	0.411
Females	4.45 ± 0.86	4.34 ± 0.86	0.419
Males	4.77 ± 0.77	4.7 ± 0.83	0.741
Glucose (mg/dL)	78.56 ± 10.71	78.08 ± 10.43	0.746
Females	78.67 ± 10.81	78.49 ± 10.33	0.900
Males	78.19 ± 10.52	76.71 ± 10.88	0.625
LDL (mg/dL)	105.17 ± 25.32	103.22 ± 25.45	0.599
Females	102.78 ± 25.58	100.68 ± 25.41	0.611
Males	112.97 ± 23.14	111.57 ± 24.31	0.779
HGB (g/dL)	13.55 ± 0.85	13.40 ± 0.89	0.189
Females	13.53 ± 0.86	13.36 ± 0.89	0.223
Males	13.63 ± 0.83	13.51 ± 0.91	0.704
PLT (10^3^/μL)	228.33 ± 54.46	221.87 ± 46.10	0.563
Females	224.46 ± 53.89	220.64 ± 47.56	0.824
Males	240.89 ± 55.17	225.9 ± 41.78	0.422
CRP (mg/L)	3.06 ± 1.99	3.27 ± 1.91	0.206
Females	2.93 ± 1.91	3.23 ± 1.99	0.179
Males	3.49 ± 2.22	3.4 ± 1.66	0.934
WBC (10^3^/μL)	6.96 ± 1.11	6.66 ± 1.05	0.039*
Females	6.99 ± 1.11	6.75 ± 1.09	0.137
Males	6.86 ± 1.13	6.39 ± 0.85	0.105
Monocyte (cell/μL)	592.35 ± 80.38	463.44 ± 57.85	<0.001*
Females	587.86 ± 75.96	460.43 ± 56.63	<0.001*
Males	606.94 ± 93.01	473.33 ± 62.08	<0.001*
HDL (mg/dL)	45.34 ± 5.33	54.16 ± 4.78	<0.001*
Females	45.47 ± 5.21	54.16 ± 5.07	<0.001*
Males	44.92 ± 5.73	54.14 ± 3.8	<0.001*
Monocyte/HDL ratio	13.39 ± 3.28	8.63 ± 1.39	<0.001*
Females	13.22 ± 3	8.58 ± 1.39	<0.001*
Males	13.97 ± 4.07	8.79 ± 1.42	<0.001*
Triglyceride (mg/dL)	117.86 ± 56.21	100.87 ± 46.09	0.030*
Females	116.02 ± 51.75	105.01 ± 48.34	0.158
Males	123.86 ± 69.28	87.24 ± 35.41	0.028*

*p < 0.05 indicates statistically significant difference. Bold values indicate statistically significant results (*p* < 0.05).

### Clinical and laboratory parameters according to Fazekas score

Migraine patients were categorized into three groups based on WMH severity (Fazekas 0, 1, and 2). No significant differences were found among these groups in terms of age, BMI, lymphocyte/neutrophil counts, glucose, LDL, WBC, hemoglobin, platelet count, CRP, or headache duration (*p* > 0.05). However, monocyte count, HDL, MHR, and aura status differed significantly across the groups (*p* < 0.05) (Table 2).

**Table 2:** Clinical and laboratory parameters by Fazekas score.

Parameter	Fazekas 0 (*n* = 101)	Fazekas 1 (*n* = 40)	Fazekas 2 (*n* = 12)	*p*-value
Monocyte (cell/μL)	550.89 ± 39.60	658.75 ± 76.26	720.00 ± 69.41	<0.001*
HDL (mg/dL)	47.62 ± 2.51	41.98 ± 6.72	37.33 ± 3.92	<0.001*
Monocyte/HDL ratio	11.60 ± 1.06	16.07 ± 3.03	19.53 ± 3.12	<0.001*
Aura Presence (%)	10.9	32.5	50.0	<0.001*
Headache Duration (years)	7.05 ± 2.83	8.15 ± 3.25	6.92 ± 3.20	0.133
Age (years)	29.62 ± 5.25	29.90 ± 5.35	30.33 ± 5.96	0.880
BMI (kg/m^2^)	24.14 ± 2.67	24.96 ± 2.67	24.43 ± 1.72	0.234
Lymphocyte (10^3^/μL)	2.27 ± 0.99	2.39 ± 0.83	2.40 ± 1.16	0.707
Neutrophil (10^3^/μL)	4.48 ± 0.87	4.53 ± 0.86	4.87 ± 0.56	0.418
Glucose (mg/dL)	78.75 ± 10.81	79.50 ± 10.71	73.75 ± 9.32	0.247
LDL (mg/dL)	104.39 ± 25.97	105.96 ± 25.87	109.14 ± 18.20	0.890
WBC (10^3^/μL)	6.93 ± 1.15	6.99 ± 1.02	7.14 ± 1.14	0.860
HGB (g/dL)	13.51 ± 0.86	13.65 ± 0.90	13.58 ± 0.59	0.658
PLT (10^3^/μL)	229.31 ± 54.55	221.98 ± 53.27	241.25 ± 59.43	0.506
CRP (mg/L)	3.23 ± 2.03	2.53 ± 1.78	3.42 ± 2.19	0.154

*p < 0.05 indicates statistically significant difference.

Monocyte count and MHR were positive correlated with Fazekas scores (*p* < 0.001, *r* = 0.496). HDL levels were negative correlated with Fazekas scores (*p* < 0.001, *r* = −0.512). Aura was more common in patients with higher Fazekas scores (*p* < 0.001).

### WMH presence vs. absence

Patients were divided into WMH-negative (Fazekas 0) and WMH-positive (Fazekas 1 + 2) groups. No differences were observed in age, BMI, headache duration, lymphocyte/neutrophil counts, glucose, LDL, triglycerides, WBC, hemoglobin, platelet count, or CRP (*p* > 0.05). However, monocyte count, HDL, MHR, and aura status showed significant differences (*p* < 0.05) (Table 3).

**Table 3:** Clinical and laboratory characteristics of migraine patients with and without lesions.

Parameter	No lesion (*n* = 101)	Lesion present (*n* = 52)	*p*-value
Monocyte (cell/μL)	550.89 ± 39.60	672.88 ± 78.52	<0.001*
HDL (mg/dL)	47.62 ± 2.51	40.90 ± 6.46	<0.001*
Monocyte/HDL ratio	11.60 ± 1.06	16.87 ± 3.35	<0.001*
Aura presence (%)	10.9	36.5	<0.001*
Headache duration (years)	7.05 ± 2.83	7.87 ± 3.25	0.121
Age (years)	29.62 ± 5.25	30.00 ± 5.44	0.652
BMI (kg/m^2^)	24.14 ± 2.67	24.84 ± 2.48	0.102
Lymphocyte (10^3^/μL)	2.27 ± 0.99	2.39 ± 0.90	0.405
Neutrophil (10^3^/μL)	4.48 ± 0.87	4.61 ± 0.81	0.446
Triglyceride (mg/dL)	115.79 ± 58.48	121.88 ± 51.85	0.364
Glucose (mg/dL)	78.75 ± 10.81	78.17 ± 10.60	0.752
LDL (mg/dL)	104.39 ± 25.97	106.70 ± 24.19	0.683
WBC (10^3^/μL)	6.93 ± 1.15	7.02 ± 1.04	0.703
HGB (g/dL)	13.51 ± 0.86	13.63 ± 0.84	0.372
PLT (10^3^/μL)	229.31 ± 54.55	226.42 ± 54.77	0.793
CRP (mg/L)	3.23 ± 2.03	2.73 ± 1.89	0.171

*p < 0.05 indicates statistically significant difference.

WMH-positive patients had higher monocyte counts and MHR (*p* < 0.001). HDLwas lower in WMH-positive patients (p < 0.001). Aura was more frequent in the WMH-positive group (*p* < 0.001).

### Receiver operating characteristic analysis

**Figure 1:**
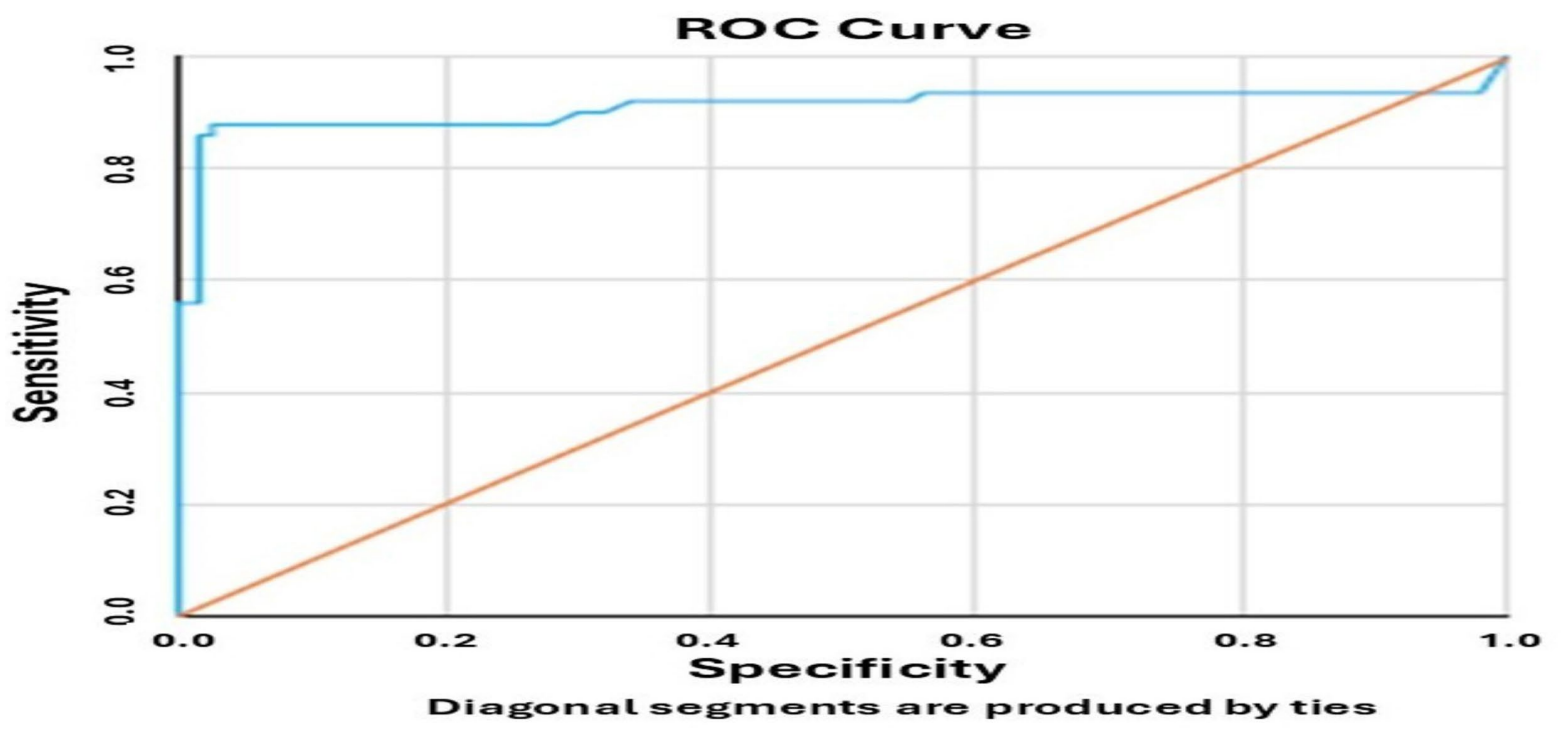
Receiver operating characteristic (ROC) curve showing the predictive accuracy of the monocyte-to-HDL cholesterol ratio (MHR) for the presence of white matter hyperintensities (WMH). The blue curve represents the diagnostic performance of the MHR in predicting WMH, while the diagonal orange line indicates the no-discrimination reference line (chance level). The optimal cut-off point was 13.37, the area under the curve (AUC) was 0.918 (95% confidence interval: 0.851–0.984; *p* < 0.001), the sensitivity was 88.50%, and the specificity was 98.00%.

An ROC analysis was performed to assess the predictive value of the monocyte-to-HDL cholesterol ratio (MHR) for WMH presence. The ROC curve (Figure 1) demonstrated an area under the curve (AUC) of 0.918 (95% confidence interval: 0.851–0.984; *p* < 0.001), with a sensitivity of 88.50% and specificity of 98.00% at an optimal MHR cut-off point of 13.37. These results suggest that MHR may serve as a reliable biomarker for identifying WMH in migraine patients.

### Logistic regression analysis

Longer disease duration was significantly associated with Fazekas 1 grade. Higher MHR levels were significantly associated with increasing WMH severity. Age and sex were also included as covariates in the regression analyses but did not reach statistical significance. Both binary and multiple logistic regression models were statistically significant (*p* < 0.01) (Table 4).

**Table 4:** Analysis of regression models according to the Fazekas scale in migraine patients.

Variable	βeta	Exp (B) (95% CI)	*p*-value
(1) Binary logistic regression analysis for white matter lesion presence/absence			
Age	−0.007	0.993 (0.862–1.145)	0.925
Sex (ref. female)	0.650	1.916 (0.436–8.430)	0.390
Aura (ref. absence)	0.944	2.570 (0.449–14.703)	0.289
BMI	0.076	1.079 (0.846–1.378)	0.539
M/HDL ratio	1.164	**3.204 (2.151–4.772)**	**<0.001**
Ilness duration	0.348	**1.416 (1.099–1.824)**	**0.007**
Constant	−20.915	0.000	**<0.001**

(2) Multinominal logistic regression analysis for white matter lesion 0.1 or 2			
Fazekas 1			
Age	−0.008	0.992 (0.860–1.145)	0.915
Sex (female)	−0.662	0.516 (0.117–2.274)	0.382
Aura (absence)	−0.988	0.372 (0.065–2.130)	0.267
BMI	0.082	1.085 (0.850–1.386)	0.511
M/HDL ratio	**1.130**	**3.095 (2.073–4.621)**	**<0.001**
Ilness duration	**0.354**	**1.424 (1.104–1.837)**	**0.006**
Fazekas 2			
Age	0.035	1.036 (0.829–1.295)	0.757
Sex (female)	−0.560	0.571 (0.060–5.401)	0.625
Aura (absence)	−0.173	0.841 (0.063–11.208)	0.896
BMI	−0.022	0.978 (0.668–1.432)	0.910
M/HDL ratio	**1.598**	**4.942 (2.796–8.735)**	**<0.001**
Ilness duration	**0.261**	**1.298 (0.874–1.929)**	0.196

Hosmer and lemeshow: chi-square: 22.139 and *p*: 0.005.

−2 log likelihood: 69.010 and Nagelkerke *R*^2^: 0.78.1.

Omnibus tests: chi-square: 127.121 and *p*: <0.001.

Model fitting: chi-square: 139.880 and *p* < 0.001.

Goodness of fit: Pearson: 0.016 and deviance: 1.00.

Nagelkerke *R*^2^: 0.742.

*p < 0.05 indicates statistically significant difference. Bold values indicate statistically significant results (*p* < 0.05).

## Discussion

This study examined the association between the monocyte-to-HDL cholesterol ratio (MHR) and white matter hyperintensities (WMHs) in migraine patients. Our results demonstrated that MHR was significantly higher in migraine patients compared to healthy controls and a positive correlation was observed with both the presence and severity of WMHs. Higher Fazekas scores were associated with increased MHR values, indicating a potential link between systemic inflammation and cerebral microangiopathy in migraine.

These findings are consistent with Ulusoy et al. (18) who reported a positive correlation between MHR and WMHs in migraine patients. Similarly, Nam et al. (19) reported an association between elevated MHR and cerebral small vessel disease markers, including WMHs, lacunes, and microbleeds. Our findings extend this association to a younger cohort (mean age ~29.5 years), suggesting that inflammation-related cerebral changes may occur even in early adulthood. Additionally, Schramm et al. (22) reported that in an older population-based cohort (mean age 60.9 ± 13 years), a significant association between headache history and WMH volume was observed in women but not in men. In contrast, our cohort was significantly younger (migraine: 29.7 ± 5.3 years; control: 29.0 ± 5.2 years) and free of vascular comorbidities such as hypertension, diabetes, or cardiovascular disease; these were applied as exclusion criteria rather than statistical covariates. Sex-stratified analyses in our sample showed a significantly higher prevalence of WMHs in both female and male migraineurs compared with controls. These findings suggest that, in contrast to the female-specific association observed by Schramm et al. (22) migraine may be associated with increased WMH burden in both sexes in younger, healthy populations. However, the absence of WMHs in our healthy control group limits our ability to disentangle the relative contribution of gender and migraine to WMH occurrence, and future studies with larger participants and more diverse cohorts are required.

In contrast, Tekeşin and Tunç (23) did not reported a significant association between MHR and WMHs in migraine. However, methodological limitations, including the absence of Fazekas grading or confounder adjustment, may explain these results.

Monocytes contribute to inflammation and endothelial activation, while HDL has anti-inflammatory and antioxidant features (24, 25). Thus, an elevated MHR represents a pro-inflammatory state conducive to cerebral microvascular damage (18, 19). It’s important to note that elevated MHR may not be specific to migraine. Previous studies have shown increased MHR levels in systemic inflammatory and cardiometabolic disorders, particularly rheumatoid arthritis, which is associated with insulin resistance and cardiovascular risk, and gout, which is characterized by chronic inflammation. Therefore, elevated MHR in migraineurs may reflect not only migraine-related mechanisms but also broader systemic inflammatory activity (16, 17).

Longitudinal studies have shown that WMHs in migraine patients may progress over time (26–29). Identifying MHR as a marker of WMH burden may enable early detection of microvascular involvement and inform long-term management strategies.

The strengths of this study are as follows: First, the use of Fazekas-based MRI evaluation; second, exclusion of major comorbidities, and comprehensive inflammatory diseases. Third, WMH-related inflammation was evident even in young adults without overt systemic disease.

### Limitations of study

First, the cross-sectional design, which precludes causal inferences. Second, unmeasured factors such as diet or genetics may influence MHR levels. Additionally, lesion location, morphometric features, and the presence of cerebral microbleeds or lacunes were not analyzed and broader inflammatory markers (e.g., IL-6, TNF-*α*) were not included. Finally, none of the healthy controls, male or female, exhibited Fazekas ≥1 WMH, likely reflecting the younger age and absence of vascular comorbidities in this group. Consequently, although we observed a significantly higher WMH burden in migraineurs of both sexes, our ability to determine the relative contribution of the migraine-sex relationship to WMH occurrence was limited. Larger, older control cohorts will be needed to clarify these sex-specific associations. The *p*-value of 0.005 obtained for Hosmer-Lemeshow in Table 4 may indicate a statistical limitation in model calibration. This finding may be related to statistical sensitivity arising from the strong association between MHR and Fazekas score and is noted as a methodological limitation. Additionally, Correction for Multiple Comparisons was not applied in this study, which is a methodological limitation.

## Conclusion

MHR is significantly associated with the presence and severity of WMHs in migraine patients. Higher Fazekas scores associated with higher MHR and monocyte levels and lower HDL levels. An MHR > 13.37 strongly predicted WMH presence with high sensitivity and specificity. These results suggest that MHR may serve as a feasible and accessible biomarker for identifying microvascular involvement in migraine, even among younger patients. Further prospective studies are needed to validate its clinical applicability.

## Data Availability

The raw data supporting the conclusions of this article will be made available by the authors, without undue reservation.
